# Substance Misuse Trajectories and Risk of Relapse in the Early Course of Bipolar Disorder

**DOI:** 10.3389/fpsyt.2021.656912

**Published:** 2021-05-04

**Authors:** Trine Vik Lagerberg, Romain Icick, Sofie Ragnhild Aminoff, Mari Nerhus, Elizabeth Ann Barrett, Thomas Doug Bjella, Stine Holmstul Olsen, Margrethe Collier Høegh, Ingrid Melle

**Affiliations:** ^1^Division of Mental Health and Addiction, Norwegian Centre for Mental Disorders Research (NORMENT), Oslo University Hospital, Oslo, Norway; ^2^INSERM UMR_S1144, Paris University, Paris, France; ^3^FondaMental Foundation, Créteil, France; ^4^Early Intervention in Psychosis Advisory Unit for South East Norway (TIPS Sør-Øst), Division of Mental Health and Addiction, Oslo University Hospital, Oslo, Norway; ^5^Norwegian Center for Mental Disorders Research (NORMENT), Institute of Clinical Medicine, University of Oslo, Oslo, Norway; ^6^Departement for Specialized Psychiatry, Division of Mental Health, Akershus University Hospital, Lorenskog, Norway

**Keywords:** bipolar disorder, substance use disorders, alcohol misuse, cannabis misuse, relapse, longitudinal, early course

## Abstract

Substance misuse is highly prevalent in bipolar disorder even in the early illness phases. However, the trajectories of misuse of different substances after treatment initiation is not well-studied. Also, knowledge on how substance misuse trajectories influence the early course of bipolar disorder is limited. We recruited 220 individuals in first treatment of bipolar disorder of which 112 participated in a 1-year follow-up study at the NORMENT center in Oslo, Norway. Misuse was defined as having scores above cut-off for harmful use on the Alcohol or Drug Use Disorders Identification Tests (AUDIT or DUDIT). We investigated rates of stopping and continuing misuse of alcohol, cannabis and other illicit substances and daily nicotine use over the follow-up period, and whether such misuse trajectories predicted the risk for affective relapse. The prevalence of cannabis misuse was reduced from 29 to 15% and alcohol misuse was reduced from 39 to 21% during follow-up. Continuing alcohol misuse significantly and independently predicted affective relapse, whereas there was no difference in relapse risk between individuals stopping alcohol misuse and never misusing alcohol. Cannabis misuse trajectories did not significantly predict relapse risk although we cannot exclude interactions with alcohol misuse. In conclusion, substance misuse decreased in the early phase of bipolar disorder treatment but should be further reduced with interventions specifically addressing substance misuse. Stopping alcohol misuse is likely to yield substantial benefit on the clinical course of bipolar disorder.

## Introduction

Around half of individuals with bipolar disorder (BD) develop cannabis-, alcohol-, or other substance use disorders during their lifetime (1–3). While necessary to describe the full range of substance use levels and -patterns, the diversity in thresholds and definitions used in the field may challenge dissemination. In the current study, “use” refers to any use of substances, “use disorders” refer to substance use meeting formal diagnostic criteria, while “misuse” is used as an umbrella term also covering other definitions of potentially harmful substance use. There are indications that substance misuse is often present already at the onset of—or during the early phases of BD (4). In a recent first episode mania study, lifetime cannabis use disorder (CUD) was found in 34%, alcohol use disorder (AUD) in 15%, and other illicit substance use disorders (other SUD) in 11% of the participants (5). However, it is not well-known whether the rates of substance misuse decrease, increase or is stable during the early phases of BD. In one of the relatively few studies to date following BD individuals after their first manic episode, the proportion with alcohol misuse was found to be stable over the first 5 years of illness; 17.6% at baseline and 18.4% at follow-up. Misuse of illicit substances increased slightly from 45.9 to 52.6% (6). However, this study only investigated individuals with bipolar I disorder (BD I) and did not specifically report rates of cannabis misuse which is the most used illicit substance. Thus, while highly relevant when planning early intervention strategies, the different trajectories of misuse in representative populations of BD have been investigated to a limited extent.

Substance misuse, especially of cannabis but also of alcohol, is associated with more severe clinical characteristics in BD including earlier onset of the disorder, increased suicide risk and increased rates of rapid cycling (3, 7, 8). As these associations have mainly been established in cross-sectional studies, the current understanding of the direction of the relationship between substance misuse and BD illness severity is limited. Do individuals with BD experience more frequent episodes as a consequence of substance misuse, or are substances used more heavily as a response to symptoms in those with a more severe form of BD? This question can only be addressed through longitudinal studies, preferably during the first treatment phase where significant changes in substance misuse and clinical status are likely to take place. A previous prospective study comparing BD individuals who had never used cannabis with those who either stopped or continued their cannabis use over a 2 year period, found that those who stopped had similar relapse rates and functional outcomes to those who had never used cannabis (9). This indicates that preventing and ending cannabis (mis)use is an important clinical goal to reduce unfavorable outcomes in BD. However, the study included patients with a relatively long illness history and with low rates of cannabis use, possibly due to methodological issues such as under-reporting or sampling. In one of the few longitudinal studies on first episode mania to date, both the time in active cannabis misuse and the time in active alcohol misuse was associated with the time in affective episodes over the 5-year follow-up period (10, 11). In another longitudinal study of a first-treatment mania sample partly overlapping with the current study sample, we found that continued cannabis use was associated with higher levels of current manic symptoms and lower levels of current global functioning at 1-year follow-up compared to no use or stopped use (12). Recurrences of misuse after remission also appear to be common, further supporting the need to study misuse trajectories as a supplement to the study of lifetime comorbidity (10, 11). We have now expanded our first treatment BD sample, including participants with bipolar II disorder (BD II) and BD not otherwise specified (BD NOS). This enables a more detailed investigation of the trajectories of misuse of alcohol, cannabis and other illicit substances during the first year of treatment and their relationship to the risk of early relapse. We hypothesized that individuals with continued misuse of cannabis and/or alcohol would have significantly higher risk for relapse than patients without misuse, with intermediate risk in individuals who stopped their misuse.

## Materials and Methods

### Sample

Participants were recruited to the on-going naturalistic multi-center Thematically Organized Psychosis (TOP) Study at the NORMENT Center for Mental Health Research at Oslo University Hospital and the University of Oslo from May 2003 to November 2019. All participants provided written informed consent to participate in this study. Exclusion criteria were a history of severe head injury, IQ below 70, age outside the range of 18–65 years, and inability to give informed consent. Inclusion criteria were being in the first treatment for a primary diagnosis of BD I, II, or NOS according to the Diagnostic and Statistical Manual of Mental Disorders, 4th edition (DSM-IV) (13). First treatment was defined as giving informed consent to participate (a) within 12 months following the start of first adequate treatment or (b) while still not receiving adequate treatment. Adequate treatment was defined as taking mood stabilizing or atypical antipsychotic medication in an effective dose. Participants were not considered to be in first treatment if they previously on any occasion (before the index treatment) had received adequate treatment for more than 12 weeks. Participants who had experienced previously untreated self-remitting manic, mixed, or hypomanic episodes were also included, as were both previously, recently and never hospitalized individuals.

The TOP study is conducted in line with the Helsinki declaration of 1975 (as revised in 2008 and 2013) and has been approved by the Regional Committee for Medical Research Ethics and the Norwegian Data Inspectorate.

### Clinical Assessments

Diagnosis was established using the Structural Clinical Interview of Diagnosis for DSM-IV, Axis I disorders (SCID-I), modules A–E. The SCID-I was also used for assessment of age at illness onset and number of illness episodes. All interviewers completed a training course in SCID assessment based on the training program at UCLA (14) and participated in regular diagnostic consensus meetings led by a clinically experienced professor of psychiatry. Diagnostic reliability is assessed with regular intervals in the TOP study and has been found to be very good, with Cohen's kappa for diagnosis in the range between 0.92 and 0.99 across different assessment teams. For the main analyses, participants with BD NOS were coded as BD I if they had ever experienced manic episodes and as BD II if they had experienced hypomanic and depressive episodes. Age at onset of BD was defined as the age when the participant first met DSM-IV criteria for a major depressive, manic, hypomanic, or mixed episode. Duration of illness was calculated from age at inclusion in the study minus age at the first affective episode. Number of manic, hypomanic, mixed, and depressive episodes according to DSM-IV criteria during lifetime and during the follow-up period were recorded. The number of episodes per illness year at baseline was calculated as the total lifetime number of affective episodes divided by duration of illness. Our main variable of interest was “any relapse,” which was defined as having a new affective episode of any polarity during the follow-up period. We also explored “depressive relapse” and “(hypo)manic relapse,” which refer to having a new depressive or elevated episode during follow-up, respectively. All such episodes were defined by DSM-IV criteria. Medication use of antipsychotic agents, lithium, and antiepileptics was recorded from interview with additional information from medical records.

### Substance Use Assessments

Use of alcohol and illicit substances were assessed in-depth for each participant. Lifetime DSM-IV diagnoses of abuse or dependence of all substances were established using the SCID-I E-module. Abuse or dependence of substances other than alcohol and cannabis was compiled into a single “other substance use disorders” (other SUD) variable. The Alcohol Use Disorders Identification Test (AUDIT) (15) and the Drug Use Disorders Identification Test (DUDIT) (16) were used both at baseline and follow-up to evaluate the degree of current harmful alcohol and drug consumption, respectively. For AUDIT, scores ≥10 for males and ≥8 for females were coded as “misuse,” and for DUDIT, scores ≥3 for males and ≥1 for females were coded as “misuse.” These cut-off scores have been demonstrated as suitable for capturing substance use disorders in first episode psychosis (17). In addition, a semi-structured interview was used for assessment of recent substance use: Participants were asked whether they had used the following illicit substances: cannabis, cocaine, amphetamines, ecstasy, hallucinogens, heroin and other opiates, solvents, and non-ascribed sedatives/hypnotics during the last 6 months before baseline and follow-up assessments, as well as how many times illicit substances were used during this period. To enhance reliability, the participants were assured that no information about substance use would be shared with their clinician or others unless they explicitly gave permission. Cannabis misuse was defined as having a DUDIT score above cut-off and reporting cannabis use during the previous 6 months. DUDIT scores were missing for 13 participants at baseline and 20 participants at follow-up, and these were coded with cannabis misuse if they reported cannabis use and at least weekly use of illicit substances during the previous 6 months (*n* = 1 at baseline and *n* = 3 at follow-up). AUDIT scores were missing for 18 participants at baseline and 22 at follow-up, and these were coded as misuse if their average number of alcohol units consumed per week the previous 6 months exceeded 7 for females and 14 for males (*n* = 2 at both baseline and follow-up), in line with recommendations for maximum alcohol consumption in the Nordic countries (18). Finally, current daily use of nicotine was recorded both at baseline and follow-up since nicotine use is strongly related to all other forms of substance use or misuse (19), and has also been found to be associated with psychiatric outcomes such as suicidal risk in BD (20).

#### Substance Misuse Trajectory Groups

To investigate the relationship between misuse trajectories and relapse during the follow-up period, the sample was categorized as follows: (1) no lifetime use disorder or misuse at baseline or follow-up (NO), (2) lifetime use disorder and/or misuse at baseline but not at follow-up, i.e., stopped misuse (STOP), and (3) misuse at follow-up (with or without misuse at baseline so this group also included individuals which had started misusing) (CONT). The sample was categorized in this manner for both alcohol and cannabis misuse and in this way independent misuse trajectory variables for alcohol and cannabis were constructed.

### Statistics

Categorical data are described as counts (percentage) and continuous data as medians (interquartile range, IQR) since all continuous variables had skewed distributions according to the Shapiro-Wilk-test (all *p*-values < 0.001). Except from the initial analyses comparing the baseline characteristics of those who completed and those who dropped out of the study (as shown in Table 1), only cases which completed follow-up were included in the further analyses and data presentation. The distributions of sociodemographic and clinical variables were tested against each of the substance misuse variables (NO, STOP, and CONT for alcohol and cannabis). For bivariate analyses of continuous variables, we conducted Kruskal-Wallis *H*-tests. For categorical variables we used χ^2^-tests or Fisher's exact-tests when calculation tables for categorical variables had cells with < 5 expected cases. Statistical significance was set at *p* < 0.05, two-tailed for bivariate analyses. Significant overall effects were followed up with group-wise comparisons, which were Bonferroni corrected for multiple testing. Variables representing previously implicated predictors of relapse were examined as potential confounders in the multivariate analyses, such as age at onset (21), bipolar disorder subtype (22), frequency/number of previous episodes (23), duration of illness (24), and use of medication with mood stabilizing properties. Those significantly associated with the misuse trajectory variables (*p* < 0.05) by overall effects were entered as independent variables into hierarchic blockwise logistic regression analyses to ascertain the specific contribution of misuse trajectory on “any relapse” after controlling for potential confounders. Socio-demographic factors (if any) were entered in the first block, clinical variables including other substance related variables in the next, and substance misuse trajectories in the last block. The multivariate analyses were run twice on the whole sample to explore whether alcohol or cannabis use trajectories predicted relapse risk, and thus the level of significance was Bonferroni corrected to *p* < 0.025.

**Table 1:** Baseline sociodemographic and clinical characteristics of followed-up vs. lost to follow-up sample.

	**Followed-up (*n* = 112)**	**Lost to follow-up (*n* = 108)**	**Statistics**	
	**Median (IQR)**		**Mann-Whitney *U*-test**	***p*-value**
Age	27.0 (13.0)	25.5 (13.0)	6713.0	0.158
Education, years	14.0 (3.0)	13.0 (3.0)	5504.0	0.182
Age at onset of BD	19.0 (10.0)	18.0 (6.8)	6010.5	0.972
Duration of illness, years	7.0 (11.0)	6.0 (9.75)	6287.5	0.530
No. of affective episodes per illness year at baseline	1.0 (1.3)	1.07 (1.3)	5266.0	0.943
AUDIT at baseline^*^	7.0 (9.0)	6.0 (8.0)	5506.5	**0.039**
DUDIT at baseline^#^	0.0 (5.0)	0.0 (3.0)	5066.5	0.637
	***n*** **(%)**		**Chi**^**2**^**-test**	***p*****-value**
Gender, females, *n* (%)	69 (61.6)	62 (57.4)	χ^2^ = 0.403	0.526
Bipolar disorder type			χ^2^ = 0.540	0.764
Bipolar I disorder, *n* (%)	77 (68.8)	74 (68.5)		
Bipolar II disorder, *n* (%)	31 (27.7)	28 (25.9)		
Bipolar NOS disorder, *n* (%)	4 (3.6)	6 (5.6)		
Lithium or other mood stabilizer	50 (44.6)	49 (45.4)	χ^2^ = 0.012	0.914
Antipsychotic medication	53 (47.3)	57 (52.8)	χ^2^ = 0.655	0.418
Any adequate medication	73 (65.2)	75 (69.4)	χ^2^ = 0.454	0.500
Alcohol use disorder, *n* (%)	20 (17.9)	14 (13.0)	χ^2^ = 1.008	0.315
Cannabis use disorder, *n* (%)	12 (10.7)	14 (13.0)	χ^2^ = 0.267	0.606
Other substance use disorder, *n* (%)	9 (8.0)	9 (8.3)	χ^2^ = 0.006	0.936
Current daily nicotine use, *n* (%)	58 (51.8)	55 (50.9)	χ^2^ = 0.016	0.899

* *Twenty-six missing: 18 in the followed-up group and 8 in those lost to follow-up*.

# *Twenty-two missing: 13 in the followed-up group and 9 in those lost to follow-up*.

*IQR, Interquartile Range; BD, Bipolar Disorder; AUDIT, Alcohol Use Disorder Identification Test; DUDIT, Drug Use Disorder Identification Test; NOS, Not Otherwise Specified. Significant p-values are marked in bold*.

## Results

A total of 220 individuals with bipolar disorder (BD I *n* = 151, BD II *n* = 59, and BD NOS *n* = 10) were included in the study at baseline. Of these, 112 patients (51%) participated in a personal follow-up examination after 1 year. Of the 108 patients without data at follow-up, 20 were not planned for follow-up, 31 had moved or could not be reached, 13 had withdrawn from the study, 15 did not want to participate, 2 patients had died, and for 27 reasons were unknown. Sociodemographic and clinical characteristics of the sample at baseline, including a comparison of those who completed the study and those lost to follow-up, are presented in Table 1. The only significant difference in baseline demographic and clinical characteristics between participants who completed follow-up and those who did not out was a higher median AUDIT score in the followed-up participants (Table 1).

In the sample included in the further analyses i.e., participants who completed follow-up, median age at baseline was 27 years and median age at BD onset was 19 years. Sixty-five percent of the participants used mood stabilizing and/or antipsychotic medication, with a median age at initiation of medication of 27 years. The remaining participants had either no medication or no mood-stabilizer/antipsychotics at baseline. These were 23.5% BD I and 64.5% BD II.

### Substance Misuse at Baseline

Of the 112 participants who completed follow-up, 57 (49%) participants had misuse of alcohol, cannabis or other drugs at baseline. Thirty-two (29%) participants had cannabis misuse (including *n* = 11 with lifetime CUD). Also, one participant had lifetime CUD but no report of cannabis use the 6 months prior to baseline, indicating that the CUD was in remission. Of the 32 with cannabis misuse, 21 (19%) also had alcohol misuse. A total of 44 (39%) participants had alcohol misuse (including *n* = 20 with lifetime AUD). Also, 3 participants had lifetime AUD but AUDIT scores below cut-off at baseline, indicating that the AUD was in remission. Ten (9%) participants had misuse of other substances (including *n* = 9 with lifetime other SUD). Of these, only 2 did not have additional alcohol or cannabis misuse. Fifty-eight participants (52%) reported daily nicotine smoking at baseline. Of note, although misuse of illicit substances is present in a substantial proportion of the sample, the majority of did not have such misuse, thus the median DUDIT score at baseline is 0.0 (Table 1).

### Substance Misuse at Follow-Up

At follow-up, 33 participants (29%) had any substance misuse. Seventeen of the 32 participants with cannabis misuse at baseline had reduced their DUDIT score to below cut-off at follow-up. Two participants with reports of cannabis use increased their DUDIT score to above cut-off from baseline to follow-up. Thus, 17 participants (15%) had cannabis misuse at follow-up. Of these, 10 (9%) also had alcohol misuse. Of the 44 participants with alcohol misuse at baseline, 24 had reduced their AUDIT score to below misuse cut-off at follow-up. Four participants increased their AUDIT score to above cut-off from baseline to follow-up. Thus, 24 participants (21%) had alcohol misuse at follow-up. Of the 10 participants with other substance misuse, 4 had stopped the misuse of these substances at follow-up. Fifty-seven participants (52%-−3 with missing data) reported daily nicotine smoking at follow-up. Nine participants had stopped smoking and 9 had started smoking during the follow-up period.

**Figure 1:**
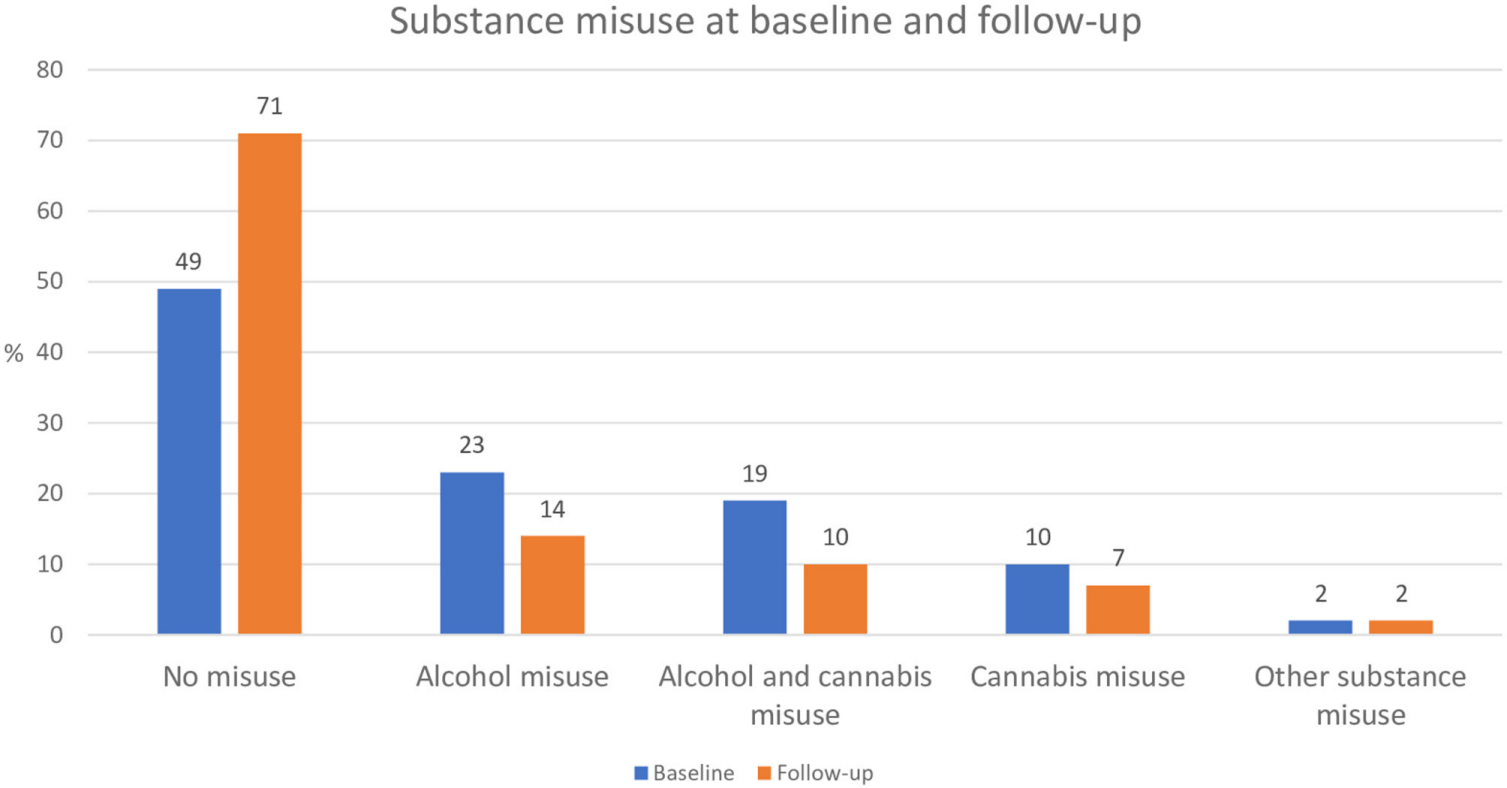
Substance misuse at baseline and follow-up (*n* = 112). Data is based on participants who completed follow-up only (i.e., baseline data on participants lost to follow-up is not included).

Rates of substance misuse at baseline and follow-up are presented in Figure 1.

### Substance Misuse Trajectories and Relapse

#### Cannabis

Sociodemographic and clinical characteristics including “any relapse” across cannabis misuse trajectories are presented in Table 2. There were no significant differences in the rate of “any relapse” between the three groups, or in the rates of depressive or (hypo)manic relapse. There were significant group differences in age at onset, and in the prevalence of alcohol misuse and daily nicotine use at follow-up.

**Table 2:** Sociodemographic and clinical characteristics across cannabis misuse trajectories.

	**NO (A)**	**STOP (B)**	**CONT (C)**	**Statistics**		
	***n* = 78**	***n* = 17**	***n* = 17**	
	**Median (IQR)**			**Kruskal Wallis *H-*test**	***P*-value**	***Post-hoc***
Age	29 (16)	24 (9)	25 (8)	*H* = 5.688	*p* = 0.061	
Age at illness onset	20 (14)	16 (4)	17 (10.5)	*H* = 8.193	***p*** **=** **0.017**	A > B
Duration of illness, years	7 (11)	9 (11.5)	6 (11.5)	*H* = 1.805	*p* = 0.406	
No. of episodes/year of illness at baseline	1.0 (1.3)	1.2 (1.4)	1.8 (1.1)	*H* = 1.838	*p* = 0.399	
	***n*** **(%)**			***X***^**2**^**/Fishers Exact-test**	***p*****-value**
Sex, female	51 (65)	10 (59)	8 (47)	*X*^2^ = 2.047, df = 2	*p* = 0.399	
Bipolar I disorder (vs. II)	59 (76)	9 (53)	13 (76)	Fisher's Exact-test = 3.576	*p* = 0.187	
Alcohol misuse at follow-up	14 (18)	8 (47)	8 (47)	Fisher's Exact-test = 11.743	***p*** **=** **0.002**	A < B, C
Other substance misuse at follow-up	2 (3)	2 (12)	2 (12)	Fisher's Exact-test = 4.509	*p* = 0.111	
Daily nicotine use at follow-up	33 (43)	10 (63)	14 (82)	*X*^2^ = 9.223, df = 2	***p*** **=** **0.010**	A < C
Antipsychotic medication at follow-up	45 (58)	9 (53)	10 (59)	*X*^2^ = 0.152, df = 2	*p* = 0.927	
Mood-stabilizer at follow-up	37 (47)	6 (35)	7 (41)	*X*^2^ = 0.930, df = 2	*p* = 0.628	
Any adequate medication	62 (80)	11 (65)	13 (76)	*X*^2^ = 1.712, df = 2	*p* = 0.425	
Depressive relapse	26 (34)	10 (59)	9 (53)	*X*^2^ = 4.686, df = 2	*p* = 0.096	
(Hypo)manic relapse	21 (28)	9 (53)	4 (27)	Fisher's Exact-test = 3.979	*p* = 0.141	
Any relapse	35 (46)	12 (71)	12 (71)	*X*^2^ = 5.687, df = 2	*p* = 0.058	

*Data are presented as median and interquartile range (IQR), and numbers and percentage*.

*NO = No misuse at baseline or follow-up, STOP = lifetime use disorder and/or misuse at baseline but not at follow-up, i.e., stopped misuse, CONT = Misuse at follow-up (with or without misuse at baseline i.e., including individuals which had started misusing). Significant p-values are marked in bold*.

To rule out a role of possible confounders in the putative relationship between “any relapse” and the cannabis misuse trajectory (*p* = 0.058), a multivariate analysis was conducted. Cannabis misuse trajectory did still not significantly predict relapse after controlling for age at BD onset, alcohol misuse and daily nicotine use at follow-up. The model was significant (χ^2^ = 15.312, df = 5, *p* = 0.009), but only alcohol misuse at follow-up was significantly associated with relapse risk (Table 3).

**Table 3:** Prediction of “any relapse” with cannabis misuse trajectories.

	***B***	**SE**	**Sig**.	**OR**	**95% CI for OR**
Age at illness onset	−0.04	0.023	0.088	0.961	0.917–1.006
Alcohol use at follow-up	1.096	0.543	**0.042**	2.994	1.033–8.673
Daily nicotine use at follow-up	−0.008	0.438	0.985	0.992	0.420–2.340
Cannabis misuse trajectory (ref. NO)					
STOP	0.640	0.675	0.343	1.896	0.505–7.116
CONT	0.559	0.636	0.380	1.749	0.502–6.087

*NO = No misuse at baseline or follow-up, STOP = lifetime use disorder and/or misuse at baseline but not at follow-up, i.e., stopped misuse, CONT = Misuse at follow-up (with or without misuse at baseline i.e., including individuals which had started misusing). Significant p-values are marked in bold*.

#### Alcohol

We then investigated the relationship between relapse and alcohol misuse trajectories. Sociodemographic and clinical characteristics including “any relapse” across the alcohol misuse trajectories are presented in Table 4. There was a significant overall effect of alcohol misuse trajectory on “any relapse.” *Post-hoc* analyses showed a higher relapse rate in the CONT group compared to both the NO group (*p* = 0.004) and the STOP group (*p* = 0.01), but no significant difference between the STOP group and the NO group (*p* = 0.892). There were also significant group differences in depressive relapse rates, and in the prevalence of misuse of other substances than alcohol and cannabis and in daily nicotine use at follow-up.

**Table 4:** Sociodemographic and clinical characteristics across alcohol misuse trajectories.

	**NO**	**STOP**	**CONT**	**Statistics**		
	***n* = 57**	***n* = 25**	***n* = 28**			
	**Median (IQR)**			**Kruskal Wallis *H-*test**	***P*-value**	***Post-hoc***
Age	30 (16)	27 (13)	24.5 (8)	*H* = 5.531	*p* = 0.063	
Age at illness onset	20 (16.5)	17 (9)	17 (7.5)	*H* = 5.694	*p* = 0.058	
Duration of illness, years	6 (10)	10 (9.5)	6 (11.75)	*H* = 2.075	*p* = 0.354	
No. of episodes/year of illness at baseline	1.2 (1.3)	1.0 (0.9)	1.0 (1.3)	*H* = 2.313	*p* = 0.315	
	***n*** **(%)**			***X***^**2**^**/Fishers Exact-test**	***P*****-value**	***Post-hoc***
Sex, female (vs. male)	32 (56)	16 (64)	19 (68)	*X*^2^ = 1.212, df = 2	*p* = 0.545	
Bipolar disorder type I (vs. II)	46 (81)	15 (60)	20 (71)	*X*^2^ = 3.931, df = 2	*p* = 0.140	
Cannabis misuse at follow-up	5 (9)	4 (16)	8 (29)	Fisher's Exact-test = 5.416	*p* = 0.060	
Other substance misuse at follow-up	0 (0)	3 (12)	3 (11)	Fisher's Exact-test = 6.660	***p*** **=** **0.015**	A < B, C
Daily nicotine use at follow-up	33 (43)	10 (63)	14 (82)	*X*^2^ = 8.230, df = 2	***p*** **=** **0.015**	A < C
Antipsychotic medication at follow-up	29 (51)	14 (56)	21 (75)	*X*^2^ = 4.554, df = 2	*p* = 0.099	
Mood-stabilizer at follow-up	30 (53)	8 (32)	12 (43)	*X*^2^ = 3.086, df = 2	*p* = 0.223	
Any adequate medication at follow-up	45 (79)	17 (68)	24 (86)	*X*^2^ = 2.470, df = 2	*p* = 0.291	
Depressive relapse	19 (33)	8 (32)	18 (64)	*X*^2^ = 8.504, df = 2	***p*** **=** **0.014**	A < C
(Hypo)manic relapse	16 (29)	7 (29)	11 (29)	*X*^2^ = 0.987, df = 2	*p* = 0.611	
Any relapse	26 (46)	11 (44)	22 (79)	*X*^2^ = 9.409, df = 2	***p*** **=** **0.009**	A, B < C

*NO = No misuse at baseline or follow-up, STOP = lifetime use disorder and/or misuse at baseline but not at follow-up, i.e., stopped misuse, CONT = Misuse at follow-up (with or without misuse at baseline i.e., including individuals which had started misusing). IQR, Interquartile Range. Significant p-values are marked in bold*.

In the multivariate analysis controlling for the potential confounders; other substance misuse and daily nicotine use at follow-up, alcohol misuse trajectory was independently associated with “any relapse” with a significantly higher relapse risk in the CONT group compared to the NO group (Table 5). The model was significant (χ^2^ = 10.867, df = 4, *p* = 0.028), with a Nagelkerke pseudo *R*^2^ of 12.9%.

**Table 5:** Prediction of “any relapse” with alcohol misuse trajectories.

	***B***	**SE**	**Sig**.	**OR**	**95% CI for OR**
Daily nicotine use at follow-up	0.249	0.426	0.558	1.283	0.557–2.955
Other substance misuse at follow-up	1.305	1.187	0.272	0.369	0.360–37.738
Alcohol misuse trajectory (ref. NO)					
STOP	−0.290	0.528	0.583	0.748	0.266–2.106
CONT	1.256	0.547	**0.022**	3.512	1.203–10.252

*NO = No misuse at baseline or follow-up, STOP = lifetime use disorder and/or misuse at baseline but not at follow-up, i.e., stopped misuse, CONT = Misuse at follow-up (with or without misuse at baseline i.e., including individuals which had started misusing). Significant p-values are marked in bold*.

## Discussion

In the current study of a first treatment BD sample, we found that the proportion of participants with any substance misuse was reduced from 49% at baseline to 29% at 1-year follow-up. We also found that participants with continued alcohol misuse were at higher risk for BD relapse during the follow-up period than participants with no history of alcohol misuse. This risk appeared to be primarily related to depressive episodes. Participants who stopped their alcohol misuse during follow-up had similar relapse risk to those who never had misused alcohol. Misuse of cannabis or other illicit substances or daily nicotine use did not independently predict the risk for BD relapse.

Participants were recruited during the initiation of the first adequate treatment, and the current study indicates that starting BD treatment contributes to reducing substance misuse. Still, one could argue that with 29% still misusing substances despite treatment initiation, additional interventions targeting substance misuse are needed. Unfortunately, we did not have any data on substance misuse-related interventions during follow-up, but participants were included from general psychiatric services where the focus on substance misuse is limited.

As different methods have been used across early phase BD studies to characterize trajectories of substance misuse, our findings are difficult to compare to previous results. Still, the misuse rates appear to vary considerably between studies, being surprisingly stable from baseline to 1-year follow-up for both alcohol and drug misuse in one study of first mania (6); while in another, rates of both cannabis and alcohol misuse were substantially reduced at 60 weeks follow-up (from 48 to 10% for cannabis misuse and from 42 to 1% for alcohol misuse) (10, 11). The rates for stopping misuse found in the current study fall somewhere in between these two studies, which may be explained by the current sample including both BD I and II disorders and previously hospitalized and non-hospitalized participants. The substantial reduction seen in the study by Strakowski et al. may for instance be due to the inpatient setting from which participants were included (10, 11), as hospitalization reduces the likelihood that patients continue substance misuse.

Somewhat contrary to expectation, we did not find significantly higher BD relapse risk in individuals with continued cannabis misuse compared to those who stopped or never misused cannabis. In bivariate analyses, there were trends for group differences in both “any relapse” and depressive relapse, but the putative association for “any relapse” appeared to be confounded by age at onset and alcohol misuse. Regarding (hypo)manic relapse, albeit not on a trend level, there was a notable numerically higher rate in those who stopped misusing cannabis compared to those who never misused or continued misusing. Although the multivariate analyses clearly indicated that the trend level for “any relapse” was driven by confounders, this is an intriguing result in the opposite direction of what one may have expected. One could speculate whether discontinuing cannabis use may trigger (hypo)mania through e.g., neuroadaptive effects or indirectly through withdrawal symptoms such as insomnia (25). The current findings of no significant differences in relapse rates between the cannabis misuse groups are in contrast to our previous finding of a relationship between continued cannabis use and higher levels of manic symptoms at follow-up (12). However, it is also possible that cannabis use induces subsyndromal symptoms rather than full-blown (hypo)manic episodes. These and other possible hypotheses should be followed up in future studies. Although some previous studies have indicated associations between cannabis use and relapse risk, longitudinal studies specifically addressing this relationship are very few. Cross-sectional studies, however, have repeatedly found associations between cannabis misuse and more severe clinical features in BD (3). The rate of alcohol misuse was however high in the group that stopped cannabis misuse, which may explain the lack of significant group differences. Indeed, when specifically addressing alcohol misuse, we found that alcohol misuse trajectory independently predicted relapse, with higher relapse rates in continued alcohol misusers compared to those with no alcohol misuse. Furthermore, there was no significant difference in relapse rates between participants with no alcohol misuse and those who stopped their alcohol misuse. This is an important clinical message, as the subsequent clinical course appears to be unaffected if alcohol misuse is stopped. However, the full model appeared to explain a modest proportion of the variance (pseudo *R*^2^ = 13%). While such a level of explanation is common in naturalistic clinical studies, these relationships need further investigation in future studies.

Of note, although there was no significant difference in rate of continued cannabis misuse across the alcohol misuse trajectories, the rate of continued cannabis misuse was relatively high in the continued alcohol misusers. One can therefore not exclude an interaction effect, i.e., that the higher relapse rate in the continued alcohol misuse group is partly explained by the continued cannabis misuse in this group. The fact that the clinical course is improved when alcohol misuse stops may indicate that individuals with substance misuse do not comprise a subgroup of BD with an underlying more severe illness form. Hence, substance misuse may elicit affective episodes and lead to a more severe BD course rather than the opposite. This hypothesis, however, needs to be further addressed in future studies.

The study has some limitations. Attrition rate was high, yielding a modestly sized sample and relatively small subgroups. Isolating the specific effects of different substances of misuse and considering the full range of misuse severity (from mild to heavy dependence) on the outcome variable is thus challenging. Also, the limited sample size hampers taking other potential confounders and risk factors into full consideration, such as comorbid anxiety disorders, personality features (e.g., impulsivity) and childhood trauma, which may also influence episode recurrence (26, 27). Although higher AUDIT scores in the participants who completed follow-up compared to those who were lost was the only significant difference between the groups at baseline, we cannot rule out that participants with misuse during follow-up were more likely to drop out of the study, which may have biased the results e.g., by inflating the reduced misuse rates at follow-up. However, individuals in the early phase of BD may be particularly difficult to retain in research, and the current study is based on one of the world's largest samples to date. Another limitation is that data were not collected regarding substance misuse-related interventions during follow-up, which would have been informative. Furthermore, the substance misuse data is based on self-report, which may be biased. However, we have previously demonstrated good correspondence between urine samples and self-reports of drug use, which is also confirmed by a meta-analysis (28, 29). The study also has several strengths. Since the sample is naturalistic and catchment area based it is likely to be representative for BD I, II and NOS individuals presenting for treatment in Norway. Furthermore, the sample is thoroughly characterized also with regards to substance use, enabling a detailed analysis of trajectories of misuse of all relevant substances. Still, there is an urgent need of further longitudinal studies to disentangle the complicated relationships between BD illness course and substance misuse, preferably with collection of more continuous and parallel data on affective symptoms and substance use.

In conclusion, this study demonstrates that the high rates of substance misuse in the early phases of BD are somewhat reduced after initiation of treatment, but also indicates that there is room for improvement in the treatment of comorbid BD and substance misuse. The risk for BD relapse over a 1-year follow-up period is higher in individuals who continue their alcohol misuse compared to individuals who have never misused alcohol, while stopping alcohol misuse appears to be of substantial clinical benefit. While the effect of alcohol misuse on the early course of BD was significant, the effect of cannabis misuse needs to be further addressed in longitudinal studies.

## Data Availability Statement

The data analyzed in this study is subject to the following licenses/restrictions: The aggregated data on each individual may be identifiable. Requests to access these datasets should be directed to Trine Vik Lagerberg, t.v.lagerberg@medisin.uio.no.

## Ethics Statement

The study was reviewed and approved by REK Sør Øst—Regional Committee for Medical and Health-related Ethics. The patients/participants provided written informed consent to participate in the study.

## Author Contributions

TL and IM designed the study. TL conducted the data analyses and drafted the manuscript. IM and RI contributed to data analyses, interpretation, and with revising the paper. SA, EB, MN, MH, and SO collected data. TB was responsible for data management and security. All authors were involved in critically reviewing the manuscript before approving the final version.

## Conflict of Interest

The authors declare that the research was conducted in the absence of any commercial or financial relationships that could be construed as a potential conflict of interest.
